# Supplementary material for: A solid‐phase transfection platform for arrayed CRISPR screens
Source: Mol Syst Biol. 2019 Dec 23;15(12):e8983. doi: 10.15252/msb.20198983 (PMC6926425; doi:10.15252/msb.20198983)
Supplement: Supplementary file 1 — Expanded View Figures PDF [file MSB-15-e8983-s001.pdf]

Expanded View Figures

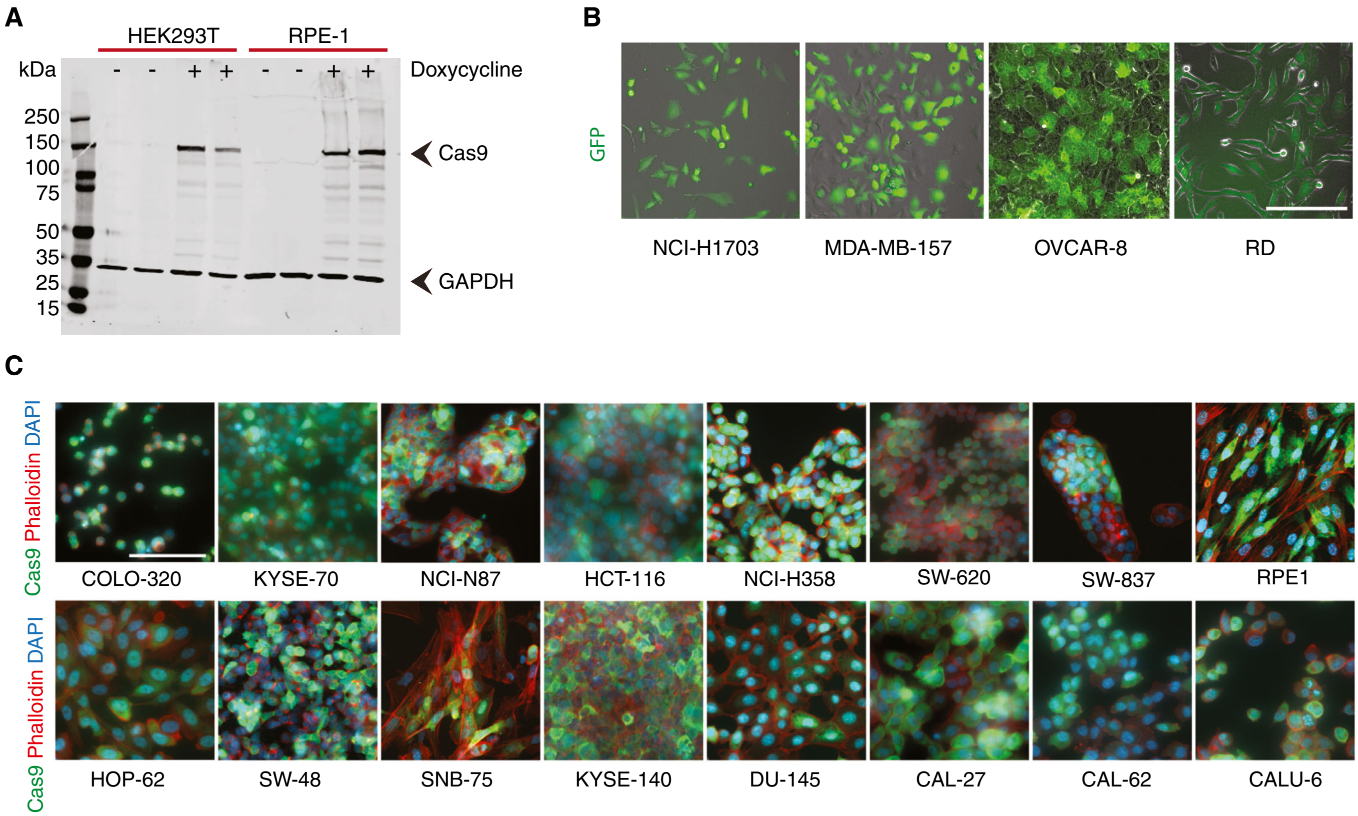

Figure EV1. Characterization of Cas9-expressing cell lines used in this study.

A Immunoblots showing inducible Cas9 expression in RPE-1 and HEK293T cell lines after 24 h of doxycycline induction (100 ng/ml).

B Cell lines stably expressing Cas9-GFP were imaged using transmitted and fluorescent light. Scale bar, 100 µm.

C Cell lines expressing inducible Cas9 were stained using anti-Cas9 (green) antibody as well as Phalloidin (red) and Hoechst (blue) to mark actin and DNA, respectively. Cells were fixed after 48 h of Cas9 induction. Scale bars, 100 µm.

 © 2019 The Authors

Figure EV2. Characterization of gRNAs used to establish the solid-phase transfection platform.

A Solid-phase transfection of nontargeting (scrambled) or *PLK1* targeting siRNA complexes into RPE-1 cells. Cells were fixed after 24, 48, and 72 h and imaged after DNA staining with Hoechst. Green arrowheads show representative cells arrested in prometaphase, and the red arrowheads show representative dead cells due to Plk1 downregulation. Scale bar, 20 μm.

B Quantification of experiments in Fig 2A and (A).

C Solid-phase transfection of *PLK1* targeting gRNAs or RNP complexes into Cas9-expressing RPE-1 or WT RPE-1 cells, respectively. Cells were lysed 24 h post-transfection, and gene editing at the relevant gene loci was assessed by Surveyor assay. Arrowheads indicate the correct size of the digested fragments by the Surveyor nuclease.

D Solid-phase transfection of *GOLGA2* targeting gRNAs or RNP complexes into Cas9-expressing RPE-1 or WT RPE-1 cells, respectively. Cells were processed and analyzed as in (C). Arrowheads indicate the correct size of the digested fragments by the Surveyor nuclease.

E Solid-phase transfection of *MKI67* targeting gRNA complexes into Cas9-expressing RPE-1 cells. Cells were fixed after 72 h, stained with Ki67 antibody (red), and analyzed by flow cytometry. GM, geometric mean of the signal intensity.

F Cas9-expressing RPE-1 cells were either mock transfected or transfected with nontargeting or *PLK1*, or *POLR2A* targeting gRNAs. Five days post-transfection, cell viability was measured by luminescent signal based on ATP production using CellTiter-Glo. The raw values are background subtracted and normalized to the untransfected controls. Results are from three independent experiments containing three technical replicates. In the boxplots, centerlines mark the medians, box limits indicate the 25th and 75th percentiles, and whiskers extend to 5th and 95th percentiles. *P* values $_{\text{(scrambled versus Plk1)}}$ and $_{\text{(scrambled versus POLR2A)}}$ < 0.001.

G Solid-phase transfection of *POLR2A* targeting gRNAs or RNP complexes into Cas9-expressing RPE-1 or WT RPE-1 cells, respectively. Cells were processed and analyzed as in (C). Arrowheads indicate the correct size of the digested fragments by the Surveyor nuclease.

H Solid-phase transfection of scrambled or *POLR2A* targeting gRNAs. Cells were fixed after 24, 48, or 72 h as indicated and stained with anti Polr2a antibody. Scale bar, 20 μm.

I Quantification of images in (H). After automated imaging and nuclear segmentation, intensity of Polr2a signal in each nucleus was measured and analyzed by ImageJ. Values are normalized to scrambled controls. Boxplots represent values from two independent experiments containing three technical replicates. Centerlines mark the medians, box limits indicate the 25th and 75th percentiles, and whiskers extend to 5th and 95th percentiles. For all time points, *P* values $_{\text{(scrambled versus POLR2A)}}$ < 0.001. Mann–Whitney *U* test.

© 2019 The Authors

© 2019 The Authors

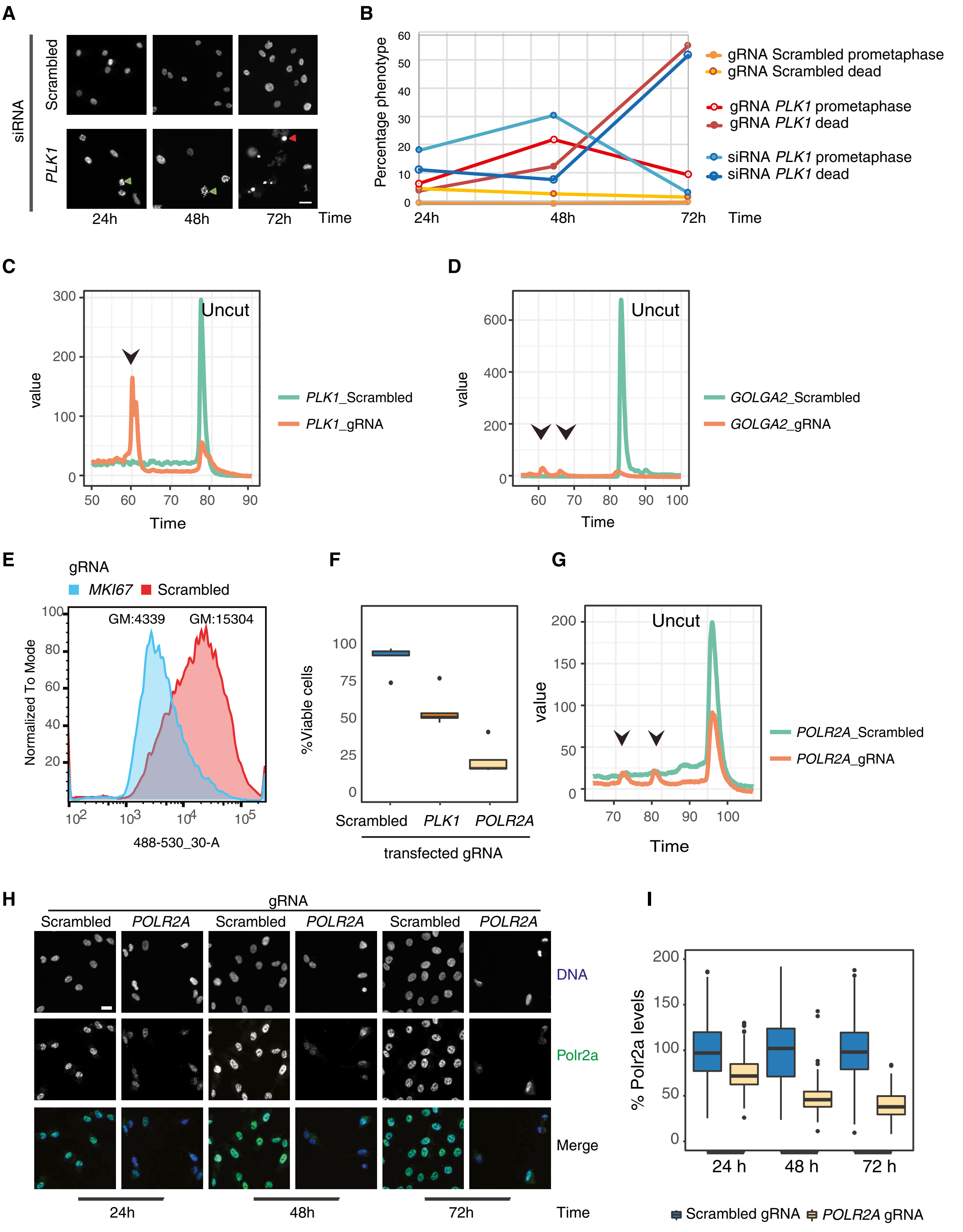

Figure EV2.

© 2019 The Authors

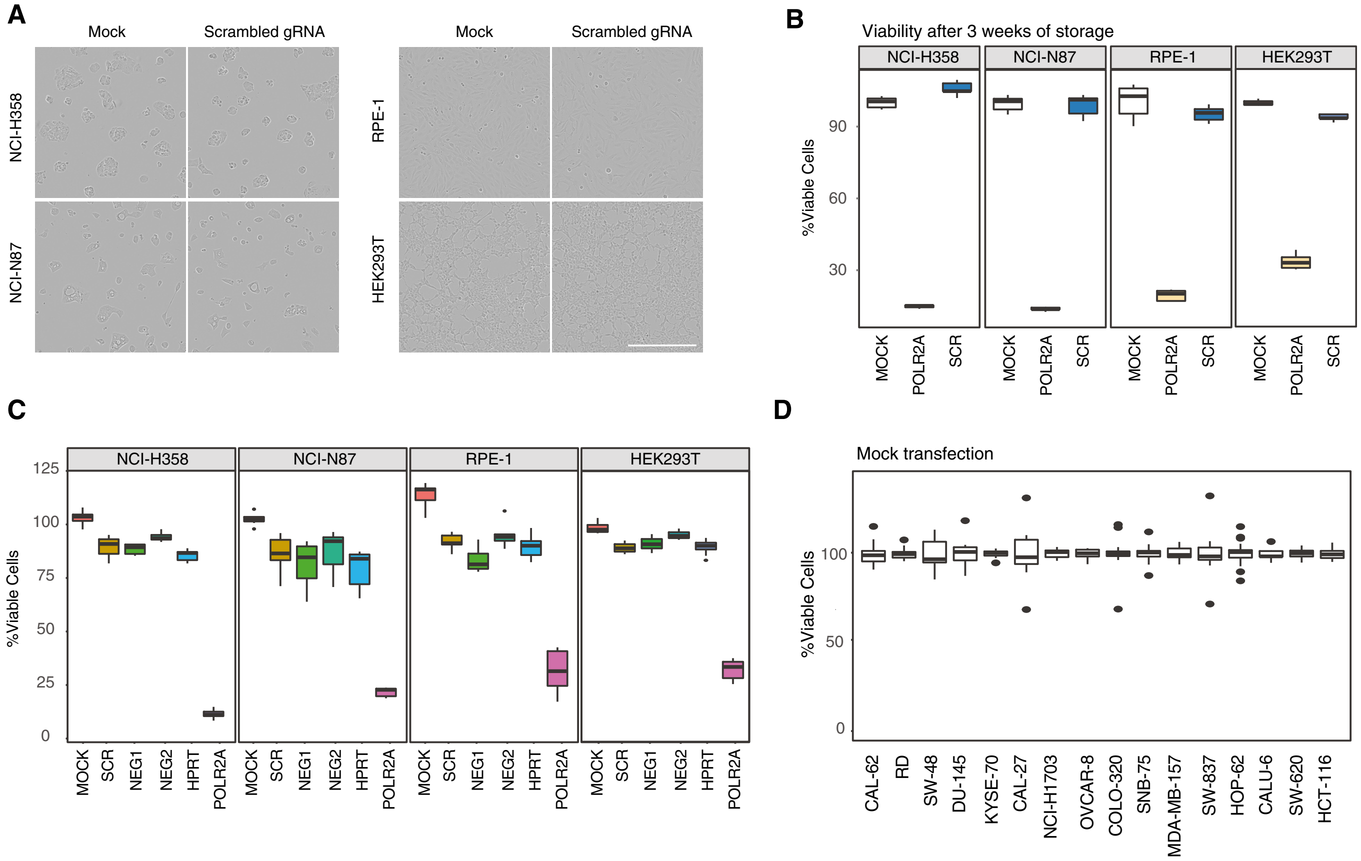

Figure EV3. Assessment of cell fitness upon solid-phase transfection.

A Solid-phase transfection of mock and scrambled gRNA in NCI-H358 and NCI-N87, RPE-1, and HEK293T cells. Transmission images of the cell lines were acquired 72 h post-transfection. Note that there are no visible fitness defects upon transfection. Scale bar, 400 µm.

B NCI-H358 and NCI-N87, RPE-1 and HEK293T cells were transfected with scrambled or *POLR2A* targeting gRNAs on plates that were stored for 3 weeks at room temperature. Five days post-transfection, cell viability was measured by CellTiter-Glo. Boxplots represent values from three independent experiments containing three technical replicates. Centerlines mark the medians, box limits indicate the 25th and 75th percentiles, and whiskers extend to 5th and 95th percentiles.

C NCI-H358 and NCI-N87, RPE-1 and HEK293T cells were transfected with indicated control gRNAs. Five days post-transfection, cell viability was measured by CellTiter-Glo. Boxplots represent values from three independent experiments containing three technical replicates. In the boxplots, centerlines mark the medians, box limits indicate the 25th and 75th percentiles, and whiskers extend to 5th and 95th percentiles.

D Cell viability measurements after mock transfection in a panel of cell lines described in Fig 2I. Boxplots represent values from three independent experiments. Centerlines mark the medians, box limits indicate the 25th and 75th percentiles, and whiskers extend to 5th and 95th percentiles.

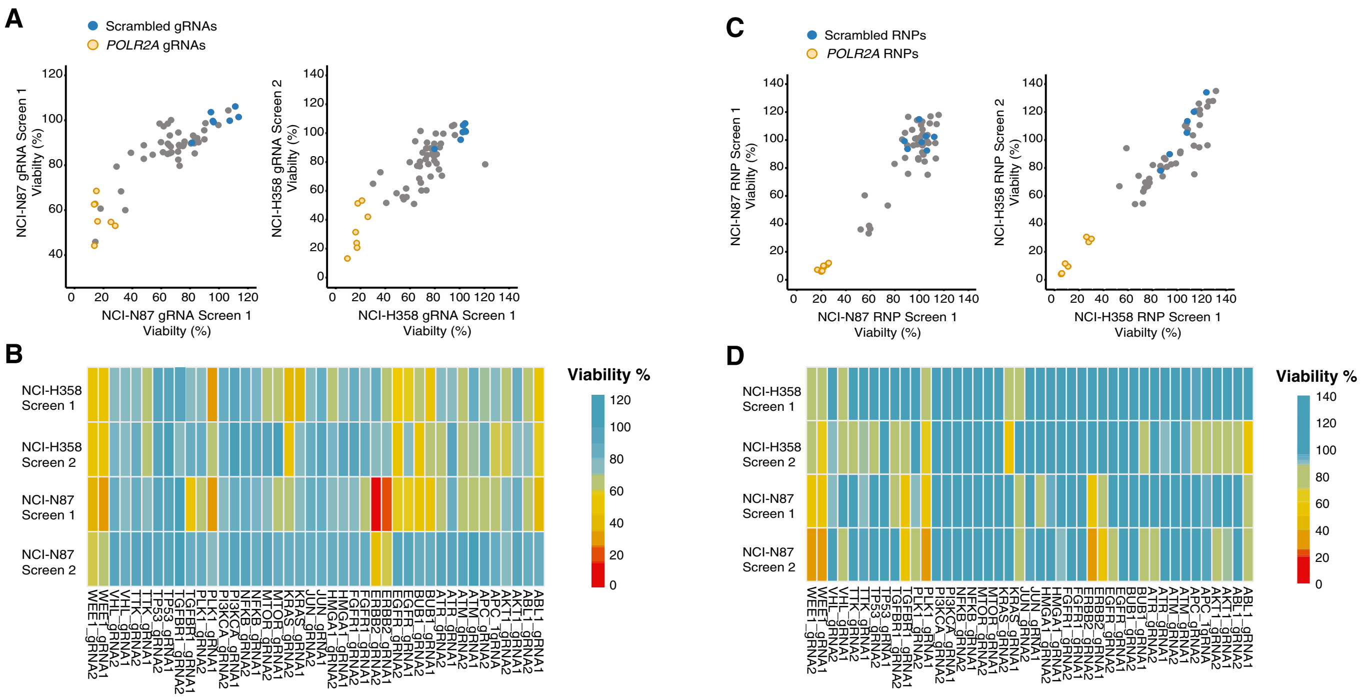

Figure EV4. Targeted gRNA and RNP screens for detecting cell line-specific oncogene addictions.

A Reproducibility of the gRNA screens described in Fig 3A and B. Viability of cells upon transfection of 45 different gRNAs by solid-phase transfection. Five days post-transfection, cell viability in each well was measured by CellTiter-Glo. Values from two independent experiments were background subtracted and normalized to nontargeting controls. Normalized values in each cell line were plotted against each other.

B Cell viability normalized to scrambled controls was plotted as heatmap for the gRNAs used in the screen.

C Reproducibility of the RNP screens described in Fig 3C. Viability of NCI-N87 and NCI-H358 cells upon transfection of 45 different RNP complexes by solid-phase transfection. Five days post-transfection, cell viability in each well was measured by CellTiter-Glo. Values from two independent experiments were background subtracted and normalized to nontargeting controls. Normalized values in each cell line were plotted against each other.

D Cell viability normalized to scrambled controls was plotted as heatmap for the RNP complexes used in the screens.

© 2019 The Authors

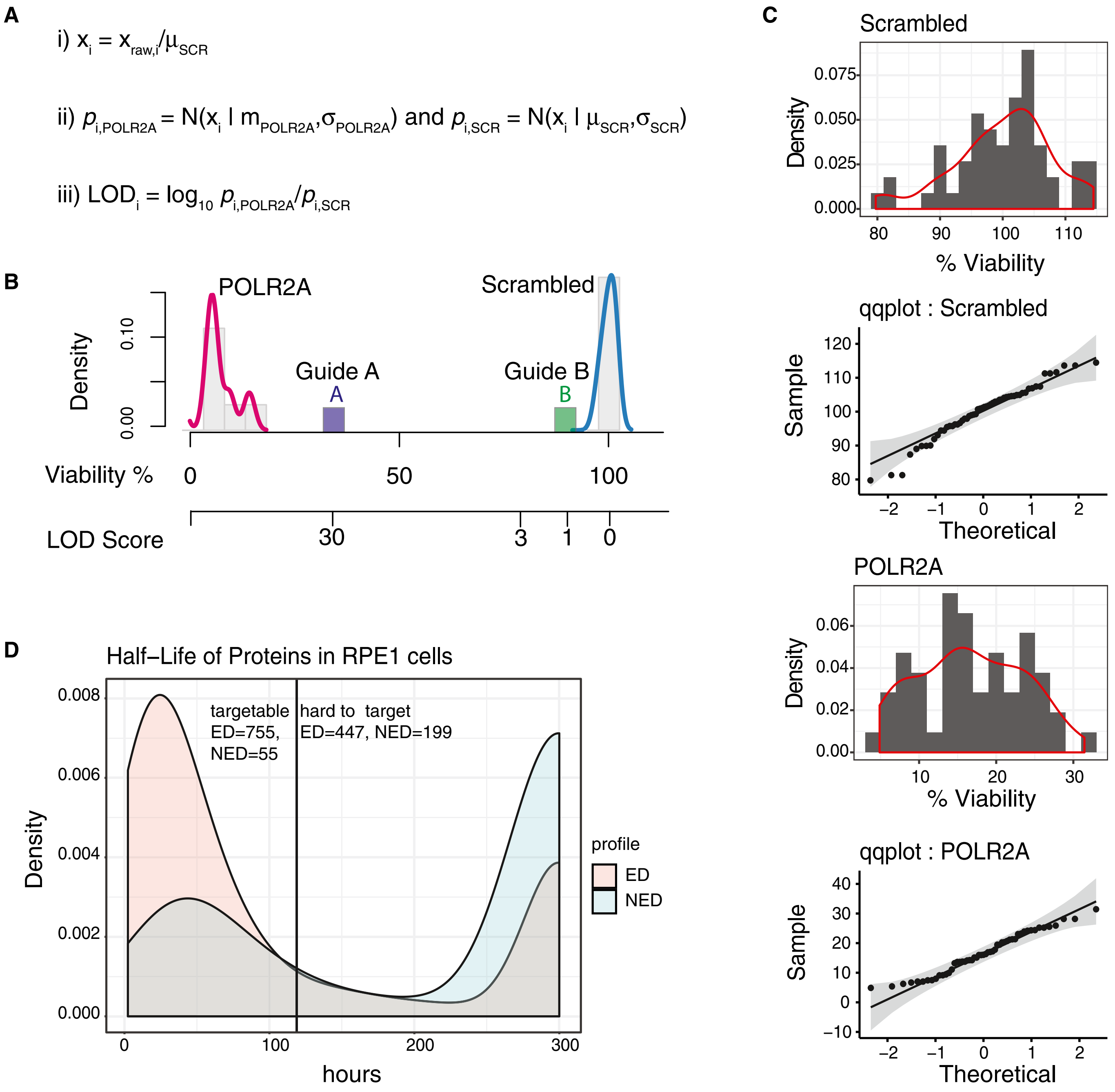

Figure EV5. Assessment of high-throughput screens based on cell viability.

A Calculation of LOD scores for effect on viability of guide RNA transfection: (i) Normalized values of viability were obtained by ratio of each guide to scrambled control transfection. (ii) Normal distribution of probabilities for each guide was calculated using cumulative normal distribution function using normalized values and mean values of controls. LOD scores were calculated for each guide using values generated in (iii) using the formula shown.

B An example of LOD score and viability graph. Density graphs show positive and negative control guides and their normal distribution. Guide A targets a gene causes strong loss of viability with a high LOD score, whereas guide B shows no loss of viability, with a low LOD score.

C Controls for normality. Distribution and qqplots of control guides used in our study. Distributions of the control guides were tested by a Kolmogorov–Smirnov test. We have not observed any significant differences to normally distributed samples.

D Prediction of proteins that can be targeted by arrayed CRISPR/Cas9-based screens. Using the dataset from McShane *et al*, we considered 1-state degradation model for exponentially degraded proteins (ED) and 2-state degradation model for non-exponentially degraded (NED) proteins. The densities of all the proteins are plotted and classified as “targetable” and “hard to target” in CRISPR/Cas9-based arrayed screens based on a threshold of 120-h half-life.

© 2019 The Authors
